# Supplementary material for: Structural and biochemical insights into small RNA 3′ end trimming by Arabidopsis SDN1
Source: Nat Commun. 2018 Sep 4;9:3585. doi: 10.1038/s41467-018-05942-7 (PMC6123492; doi:10.1038/s41467-018-05942-7)
Supplement: Supplementary file 1 — Supplementary Information [file 41467_2018_5942_MOESM1_ESM.pdf]

Supplementary Information

Structural and Biochemical Insights into Small RNA 3' End Trimming by *Arabidopsis* SDN1

Chen *et al.*

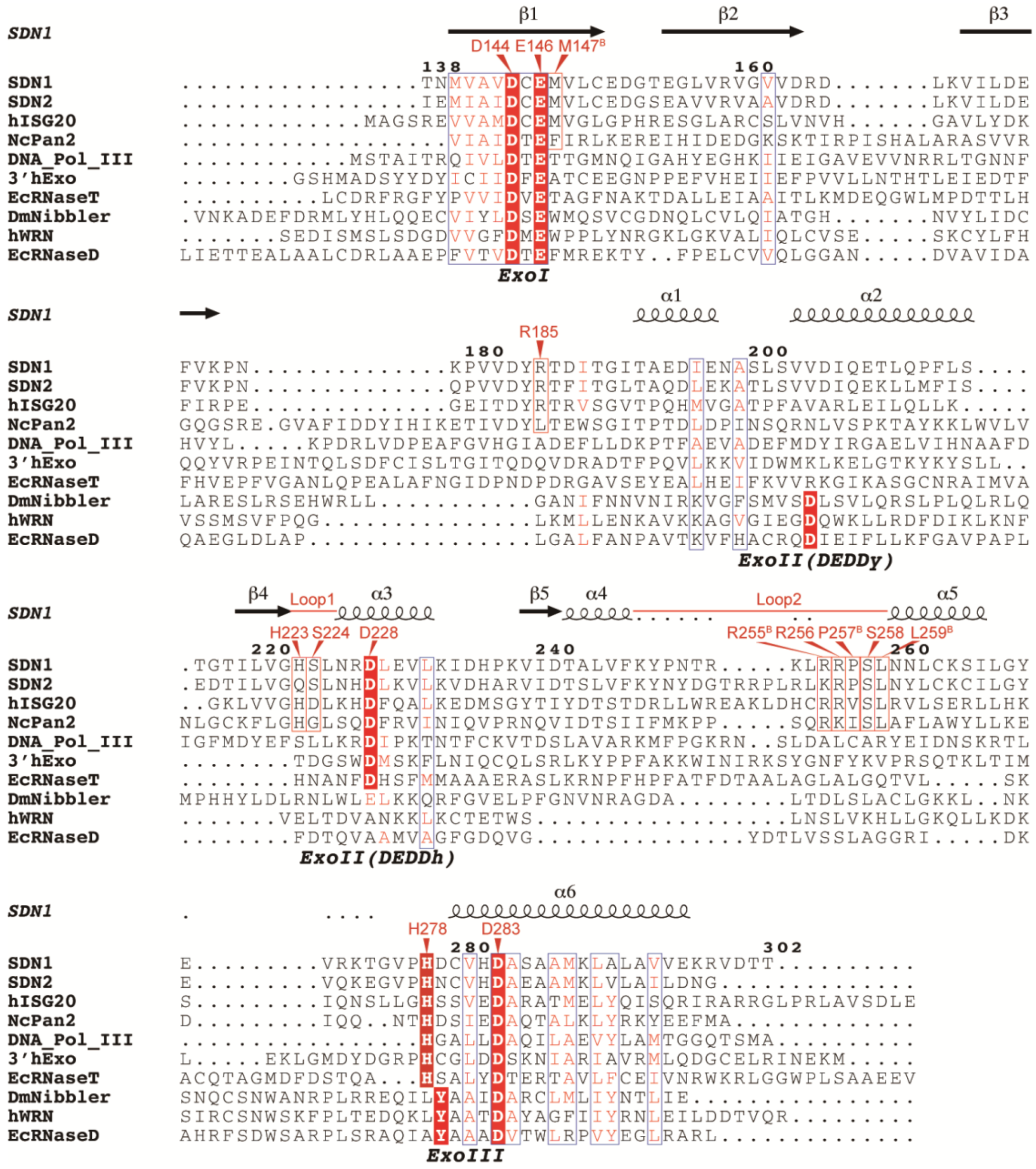

Supplementary Figure 1 Sequence alignment of the DEDDh domains of SDN1 and other exonucleases

The sequence of SDN1 DEDDh (GenBank Accession Code: OAP04471) was aligned to the following proteins: *Arabidopsis thaliana* SDN2 (GenBank Accession Code: OAO90713), *Homo sapiens* ISG20 (GenBank Accession Code: CAA61915), *Neurospora crassa* Pan2 (GenBank Accession Code: EDO65208), *Thermotoga maritima* DNA polymerase III exonuclease domain (GenBank Accession Code: AAC80438), *Homo sapiens* 3'Exo (GenBank Accession Code: AAH35279), *Escherichia coli* RNase T (GenBank Accession Code: ACI83035), *Drosophila melanogaster* Nibbler (GenBank Accession Code: Q9VIF1), *Homo sapiens* WRN (GenBank Accession Code: AAC63361) and *Escherichia coli* RNase D (GenBank Accession Code: ACI82335). Residues that participate in RNA recognition are labeled in the sequence. The sequence alignment was modified based on output files from ESPript 3.0[1].

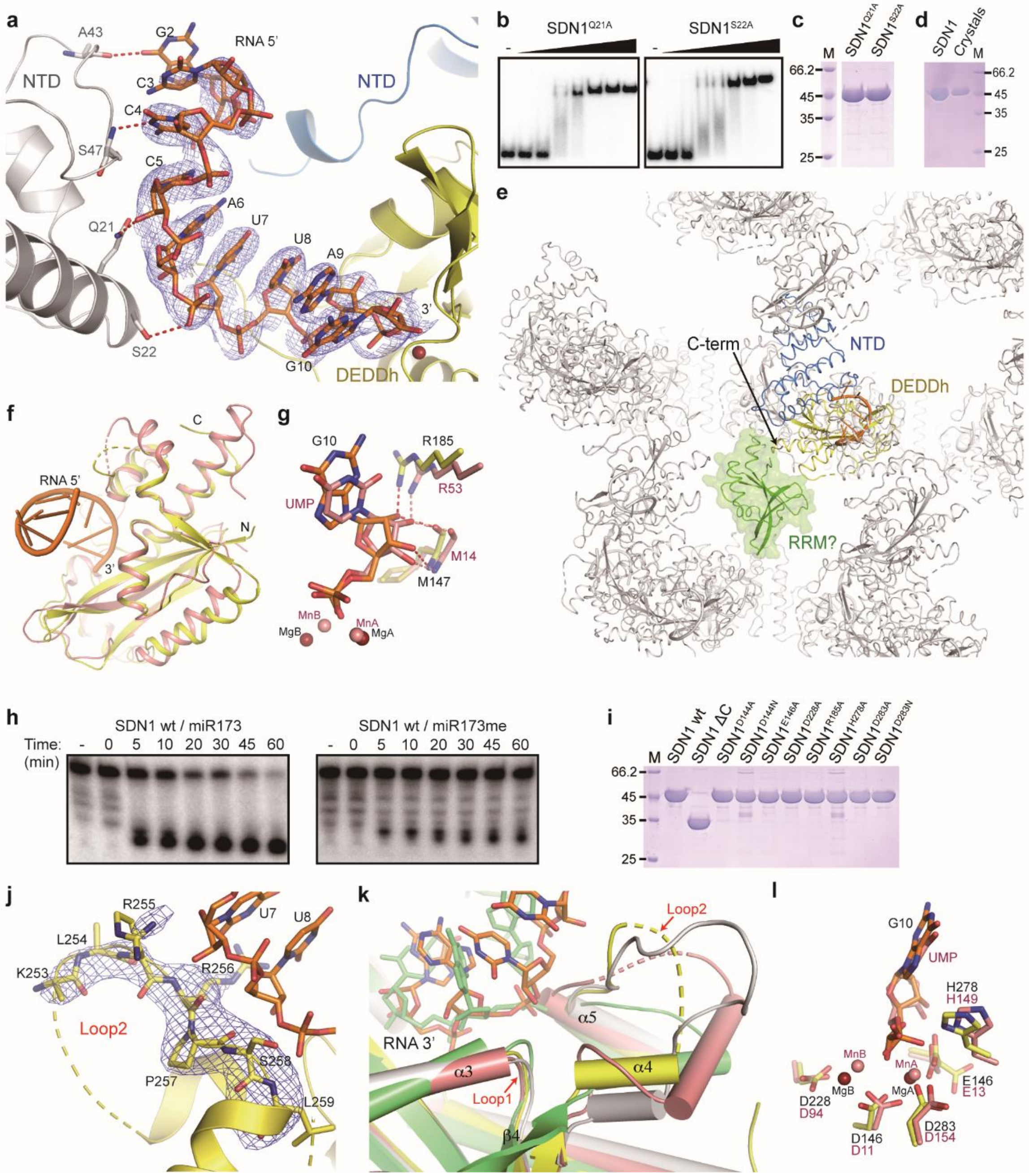

Supplementary Figure 2 Structural properties of SDN1 DEDDh and its recognition of the RNA substrate

(a) Simulated annealing Fo-Fc omit map of the RNA substrate in the structure of SDN1 ΔC-ssRNA (contoured at 2.0 σ), and the inter-molecular interactions between the RNA and the adjacent SDN1 protein (shown in grey). The interactions are hydrogen bonds between: Gln21 and the phosphate group of C5; Ser22 and the phosphate group of U7, Ala43 and the base of G2; and Ser47 and the base of C4. **(b)** EMSA assays of SDN1^{Q21A} or SDN1^{S22A} with ^{32}P-labeled miR166 show that mutagenesis of Gln21 or Ser22 does not impair the enzyme's RNA binding affinity, indicating that Gln21 and Ser22 do not take part in the enzyme-RNA interaction and the interactions in **(a)** should result from crystal packing. Assays were conducted at a series of protein concentrations: 0.5 µM, 1 µM, 5

µM, 10 µM, 50 µM, 100 µM and 500 µM (from left to right). **(c)** SDN1^{Q21A} and SDN1^{S22A} were analyzed on an SDS-PAGE gel to show the high homogeneity. **(d)** SDS-PAGE analysis of the SDN1 protein sample used for crystallization (left lane) and the SDN1 protein in the SDN1-ssRNA crystals (right lane), indicating that the SDN1 CTD was not cleaved during crystallization. **(e)** Crystal packing of the SDN1-ssRNA crystals. The coordinates of SDN1 RRM, shown in cartoon and surface representations, are placed alongside the C-terminus of the DEDDh domain to show that there is enough space in the crystal lattice to hold the C-terminal RRM domain. **(f, g)** Superimposition of the SDN1 DEDDh domain (yellow) and hISG20 (pink, PDB code: 1WLJ). The recognition of the 3' terminal ribose by Met147 and Arg185 of SDN1 and the corresponding interactions are shown in **(g)**. **(h)** *In vitro* enzymatic assays of SDN1 acting on miR173 or 3' end 2'-*O*-methylated miR173 (miR173me). Both RNAs were 5' end ^{32}P-labeled. The assays were conducted in a time course as indicated. **(i)** The SDN1 proteins used in Fig. 2f were purified to high homogeneity. **(j)** Simulated annealing Fo-Fc omit map of residues Lys253, Leu254, Arg255, Arg256, Pro257, Ser258 and Leu259, the part of loop 2 for which density could be observed (contoured at 3.0 σ level). The sidechains of Lys253 and Leu254 were not modeled due to the lack of densities. **(k)** Superimposition of the SDN1 DEDDh domain (yellow), hISG20 (pink, PDB code: 1WLJ), *Nc*Pan2 (grey, PDB code: 4CZW) and EcRNase T (light green, PDB code: 3NH2). The two loops involved in SDN1 substrate recognition are labeled. **(l)** The active site of SDN1 DEDDh (yellow) is superimposed to that of hISG20 (pink, PDB code: 1WLJ).

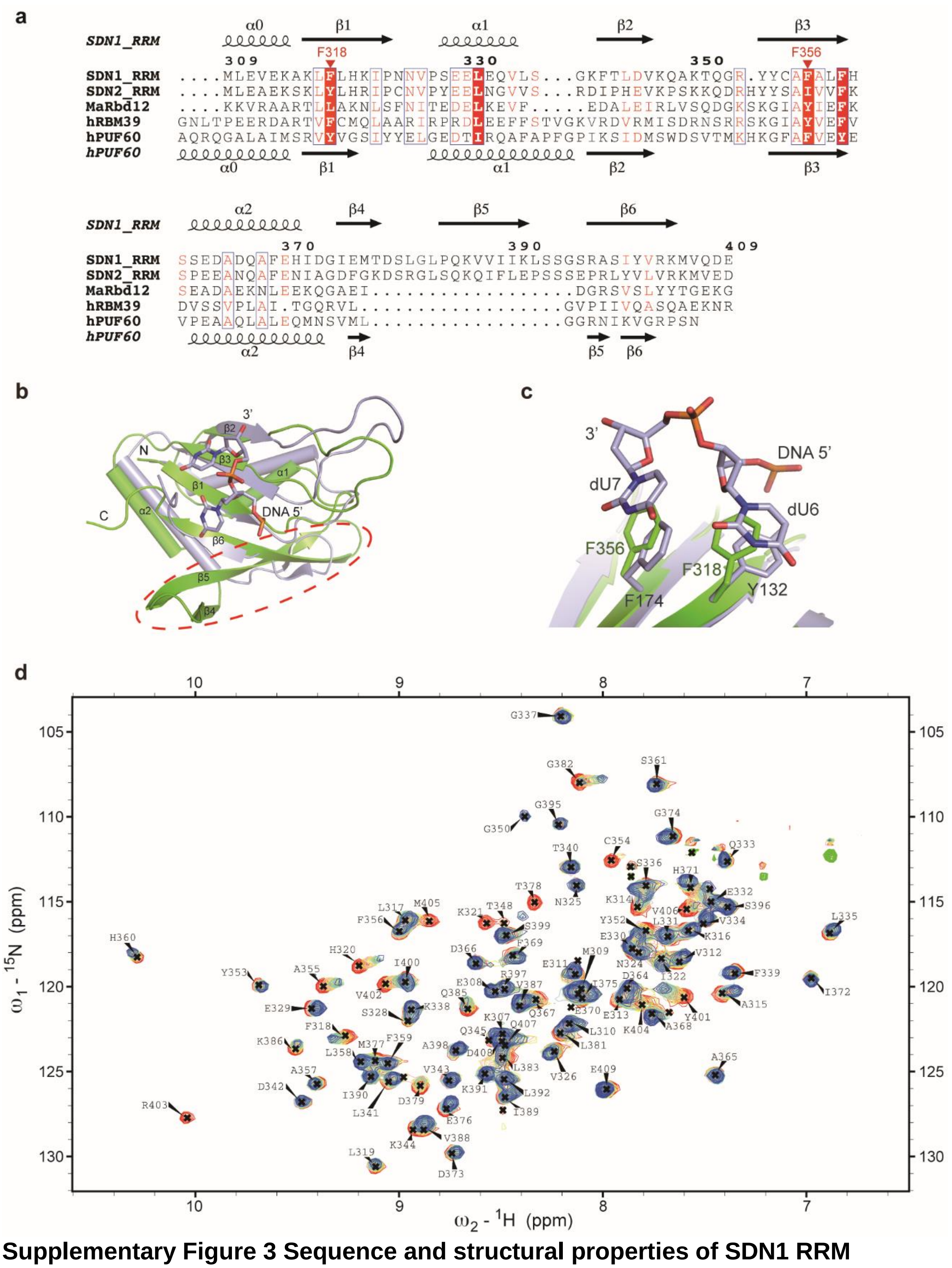

Supplementary Figure 3 Sequence and structural properties of SDN1 RRM

(a) The sequence of SDN1 RRM (GenBank Accession Code: OAP04471) was aligned to the following proteins: *Arabidopsis thaliana* SDN2 (GenBank Accession Code: OAO90713), *Mesocricetus auratus* nucleolin Rbd12 (GenBank Accession Code: 1FJE_B), *Homo sapiens* RBM39 (GenBank Accession Code: AAI31544) and *Homo sapiens* PUF60 (GenBank Accession Code: Q9UHX1). The two highly conserved aromatic residues in β1

and β3 are marked in red (Phe318 and Phe356). The sequence alignment was modified based on output files from ESPript 3.0[1]. **(b, c)** Structural superimposition of SDN1 RRM (green) and hPUF60 RRM1 (light blue, PDB code: 5KVY). The extended β4 and β5 in SDN1 RRM are circled. The two conserved phenylalanines of SDN1 RRM, Phe318 and Phe356, could be well superimposed to the corresponding amino acids of hPUF60 RRM1 **(c)**. **(d)** Overlay of the two-dimensional ^{1}H - ^{15}N HSQC spectra showing chemical shift perturbations during NMR titration. Signals were colored to represent the different protein/RNA ratio when each spectrum was recorded (red 1:0, yellow 1:0.5, pink 1:0.7, maroon 1:1.0, cyan 1:1.2, light green 1:1.5, light blue 1:1.7, and blue 1:2). Cross peaks of the residues K321, C354, T378, D379, Q385, V402, R403, K404, M405 and V406 disappeared during titration. Residues K314, A315, F318, H320, I322, Y352, A355, H371, I375, E376, G382 and Y401 showed large chemical shift perturbations.

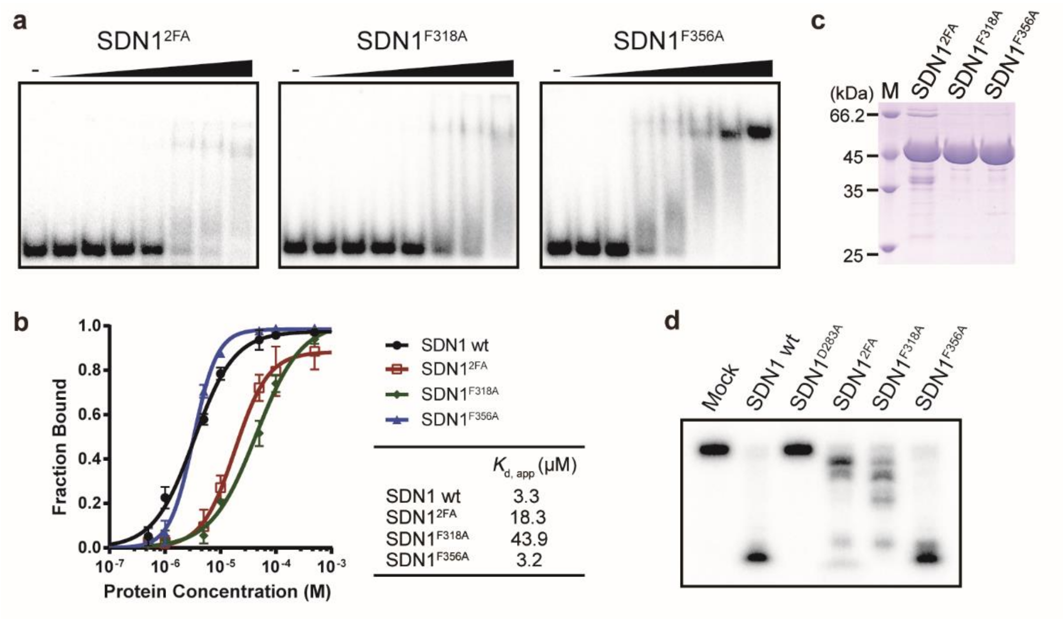

Supplementary Figure 4 Role of SDN1 RRM in enzyme-substrate interaction

(a) EMSA assays of SDN1^{2FA}, SDN1^{F318A} or SDN1^{F356A} with ^{32}P-labeled miR166. Assays were conducted at a series of protein concentrations: 0.5 µM, 1 µM, 5 µM, 10 µM, 50 µM, 100 µM and 500 µM (from left to right). **(b)** Plots showing the fraction of the protein-bound RNA at varying protein concentrations. The fraction of bound RNA was calculated from the densities of the free RNA bands in **(a)**. Scale bars represent standard deviations calculated from three biological replicates. **(c)** SDN1^{2FA}, SDN1^{F318A} and SDN1^{F356A} were purified to high homogeneity. **(d)** *In vitro* enzymatic assays of wild-type SDN1, SDN1^{D283A}, SDN1^{2FA}, SDN1^{F318A} or SDN1^{F356A} with ^{32}P-labeled miR166 in presence of 2.5 mM $MgCl_2$.

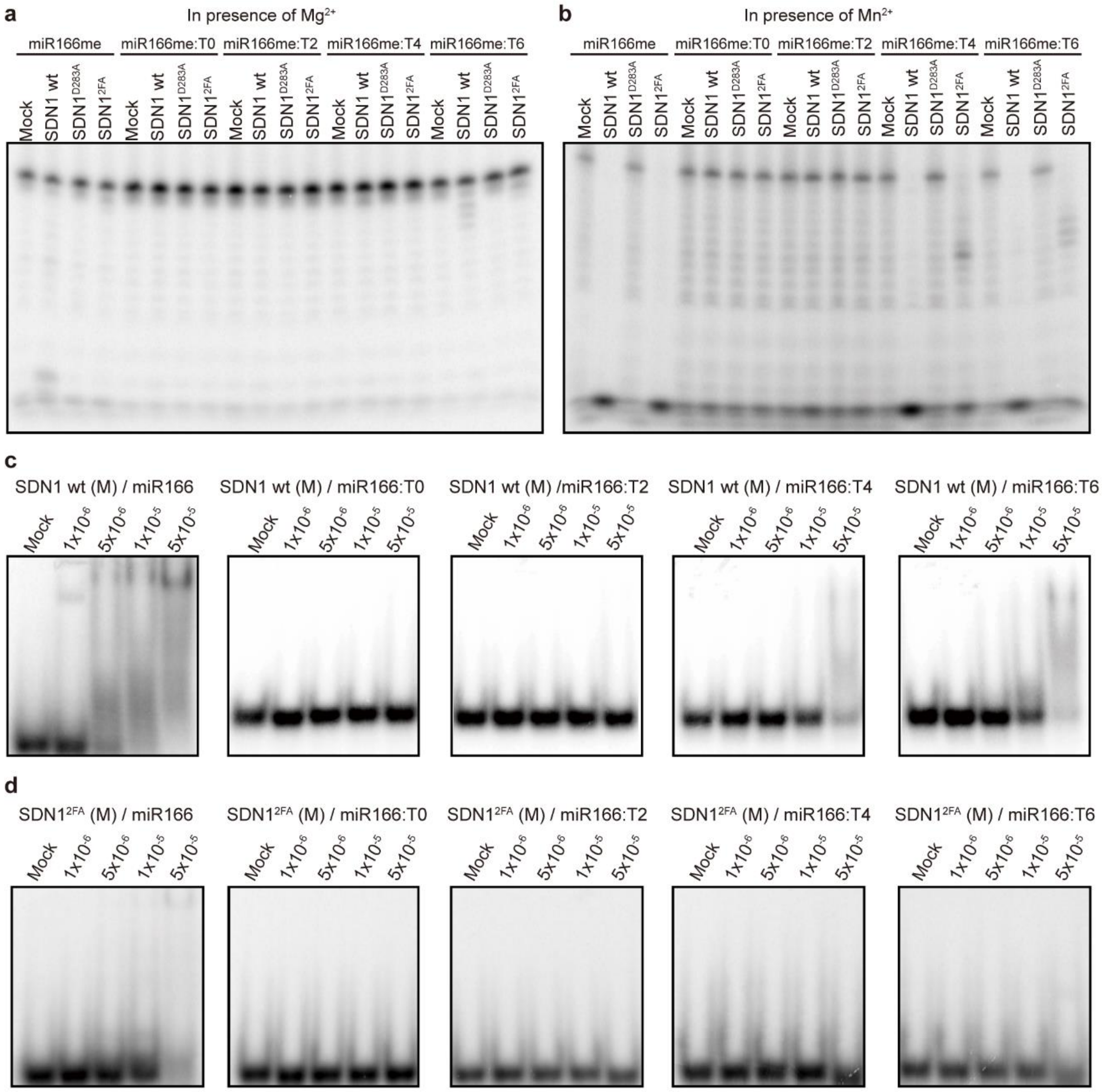

Supplementary Figure 5 The biochemical activities of SDN1 on double-stranded RNAs

(a, b) *In vitro* SDN1 enzymatic assays on 3’ end methylated miR166 in single-stranded form, or in miR166/target RNA duplex forms. The miR166 strand was 5’ end ^{32}P-labeled and monitored by denaturing polyacrylamide gel electrophoresis after the reactions with wild-type SDN1, SDN1^{D283A} or SDN1^{2FA} in the presence of Mg^{2+} **(a)** or Mn^{2+} **(b)**. **(c, d)** EMSA assays of wild-type SDN1 **(c)** or SDN1^{2FA} **(d)** with single-stranded miR166 or miR166/target RNA duplexes. Assays were conducted at a series of protein concentrations, as indicated at the top of each lane.

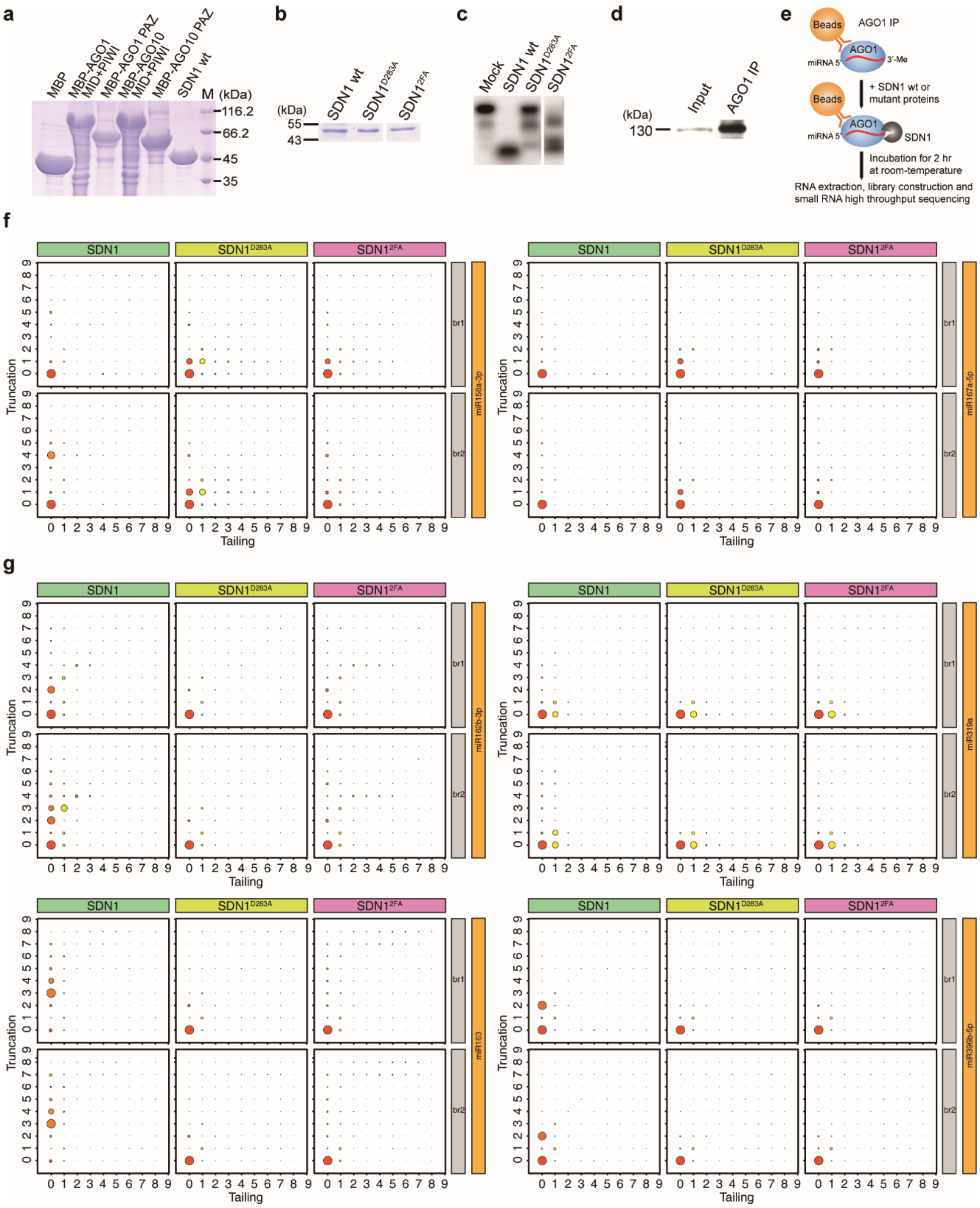

Supplementary Figure 6 ***In vitro*** **SDN1 enzymatic assays on AGO1 immunoprecipitate**

(a) SDS-PAGE analysis of the MBP-tagged bait proteins and the SDN1 target protein used in pull-down assays. **(b)** Wild-type SDN1, SDN1^{D283A} and SDN1^{2FA} were purified to high homogeneity. **(c)** *In vitro* enzymatic assays of SDN1, SDN1^{D283A} and SDN1^{2FA} on free miR166. **(d)** Western blotting with anti-AGO1 antibodies on input and AGO1 IP samples from wild-type plants. **(e)** Schematic depiction of the *in vitro* SDN1 enzymatic assays on

AGO1 IPs. **(f, g)** Diagrams showing the 3' end truncation and tailing status of several miRNAs in two biological replicates (br1 and br2). Only a few miRNAs in the SDN1^{D283A} reactions showed 1 nt truncation, exemplified by miR158a-3p and miR167a-5p **(f)**, very likely due to a contaminating nuclease. Many miRNAs were truncated by several nucleotides by wild-type SDN1, and the activities were abolished in SDN1^{D283A} and greatly reduced in SDN1^{2FA}, as exemplified by miR162b-3p, miR319a, miR163 and miR396b-5p.

Supplementary Table 1. RNAs used in biochemical assays

Name	Sequence
miR173 (22 nt RNA)	5’-UUCGCUUGCAGAGAGAAAUCAC -3’
miR173-Me	5’-UUCGCUUGCAGAGAGAAAUCAmC -3’
16nt RNA	5’- UGCAGAGAGAAAUCAC -3’
10nt RNA	5’- GAGAAAUCAC -3’
6nt RNA	5’- AAUCAC -3’
miR166	5’- UCGGACCAGGCUUCAUCCCCC -3’
T0 RNA	5’- GAGGGGGAUGAAGCUUGGUCCGGAUUA -3’
T2 RNA	5’- GAUUGGGAUGAAGCUUGGUCCGGAUUA -3’
T4 RNA	5’- GAUUCCGAUGAAGCUUGGUCCGGAUUA -3’
T6 RNA	5’- GAUUCCCCUGAAGCUUGGUCCGGAUUA -3’
3’-IUU RNA	5’- GAUUCCCUUGAAGCUUGGUCCGGA(5-IU)(5-IU)A -3’
M-IUU RNA	5’- GAUUCCCUUGAAGC(5-IU)(5-IU)GGUCCGGAUUA -3’
5’-IUU RNA	5’- GA(5-IU)(5-IU)CCCUUGAAGCUUGGUCCGGAUUA -3’

* 5-iodoU is shown as 5-IU in paratheses.

Supplementary References

1. Robert, X. & Gouet, P. Deciphering key features in protein structures with the new ENDscript server. *Nucleic Acids Res* **42**, W320-4 (2014).
